# Histopathological effects of aloe vera on wound healing process in penile fracture model: an experimental study

**DOI:** 10.3906/sag-2102-224

**Published:** 2021-08-30

**Authors:** Kadir Turgay AKGÜL, Engin DOĞANTEKİN, Elif ÖZER, Mustafa KOTANOĞLU, Yusuf GÖKKURT, Sema HÜCÜMENOĞLU

**Affiliations:** 1 Department of Urology, Ankara Training and Research Hospital, University of Health Sciences, Ankara Turkey; 2 Department of Pathology, Ankara Training and Research Hospital, University of Health Sciences, Ankara Turkey; 3 Department of Anesthesiology, Ankara Training and Research Hospital, University of Health Sciences, Ankara Turkey

**Keywords:** Penile fracture, aloe vera, wound healing, penis

## Abstract

**Background/aim:**

This study assessed the histopathological effects of aloe vera (AV) on penile fractures (PF) formed experimentally in rat model.

**Materials and methods:**

Thirty-two Wistar adult male rats (220 to 250 g) were used. The PF model was created experimentally with a number 15 lancet. After the interventions, all of the rats were randomly and equally divided into 4 groups. In the first group, incision was not closed (group C). In the second group, AV was locally applied onto incision without suturing for 3 days (group AV). In the third group, the incision line was closed primarily (group PR). In the last group, AV was applied to primary repair region for 3 days (group PAV). All groups were compared to each other according to presence of fibrosis, inflammation, and hyperemia-bleeding.

**Results:**

Hyperemia and bleeding were seen in all groups with varying degrees and the difference between groups was insignificant (p = 1.000). According to inflammation, there was a significant difference between all groups (p = 0.031). No significant inflammation was observed in group AV and therefore, group AV had a better score than group PR (p = 0.026). In group PAV, inflammation was less seen than group PR, however, the difference was insignificant (p = 0.119).According to fibrosis, group AV and group PAV had same fibrosis rates. Fibrosis was observed in 2 (25%) rats in each group. When group PR was compared with group AV and group PAV, there were no significant differences according to cavernosal tissue healing with fibrosis (p = 0.132 and p = 0.132, respectively).

**Conclusion:**

Local application of AV onto the PF region without closing with suture decreased inflammation in rats.

## 1. Introduction

Penile fracture (PF) is a rare urological emergency that is defined as a traumatic rupture of the corpus cavernosum after a high-pressure trauma to the erect penis. Masturbation and sexual intercourse are the most seen reasons in Western countries [1]. 

From its first description, PF has been managed conservatively, without surgery [2]. However, it has been observed that nearly 1/3 of the patients with PF who were treated conservatively had penis deformities and erectile dysfunction. Surgical repair, as the primary treatment modality of PF, was first presented in 1971 and today, the immediate surgical repair is the recommended treatment due to the excellent long-term results and significantly better prognosis for long-term sexual health [3–5].

Aloe vera is a medicinal plant and has been used as a medicine since 1500 before the birth of Christ in many countries. It belongs to the Liliaceae family and it is a perennial plant with thick and long leaves [6]. It contains some active substances such as anthraquinone, polysaccharides, superoxide dismutase, glycoprotein, lectin, vitamins C and E, and minerals that have antiinflammatory and wound healing effects [7–9]. Aloe vera also contains polysaccharides (e.g. glucomannans and cetylatedmannan) and monosaccharides (e.g. glucose and fructose). These components also have antiinflammatory effects [10–12]. In this study; we aimed to investigate the effect of aloe vera on wound healing at histopathological level on cavernous tissue in PF. 

## 2. Materials and methods

### 2.1. Study design

After receiving local ethics committee approval, 32 Wistar Albino male rats (220 to 250 g) were used for our study. The animals were kept in separate rat cages at 22 °C and 50% humidity and had free access to pellet food and water between January 4, 2021 and January 25, 2021.

On the study day, a prophylactic antibiotic dose (ceftriaxone, 20 mg/kg) was administered one hour before the intervention to each rat. Ketamine (50 mg/kg) (Ketalar; Parke-Davis, Detroit, MI, USA) was administered to all rats for anesthesia under sterile conditions. Then, each rat was laid in a supine position and the genital area was wiped with 10% povidone iodine after it was shaved. We placed a 3 Fr urethral catheter approximately 2 cm up to the mid urethral level from external meatus and formed a PF model with a number 15 lancet by incising from the proximal penis as described in previous studies [13,14]. All of the animals were divided into 4 groups equally and randomly. These groups were group C (control), group PR (primary repair), group AV (aloe vera), and group PAV (primary repair + aloe vera). 

In group AV, aloe vera was applied on the incision without suturing for three days. 

In group PR; incision line was closed with 6/0 polydioxanone (PDS II, Ethicon, Johnson and Johnson Ltd., India). In group PAV; incision line was closed with 6/0 polydioxanone and after the closure, aloe vera was applied incision region for 3 days. In group C, incision was not closed. We used surgical loupes (2.5×) for all procedures. At the postoperative period all of the rats urinated. After the procedures they were observed for three weeks. All animals were euthanized by intraperitoneal administration of pentobarbital sodium (100 mg/kg). The penectomies were performed from proximal parts of the incised regions with a number 15 lancet and the materials were placed separately into 10% formaldehyde solution until the day of macroscopic examination.

### 2.2. Aloe vera collection and application procedure

Aloe vera leaves were collected at the same day. We made an incision in the leaf. The mucilage of the plant was removed with a sterile swab and topically applied to incised region as described in the literature [15].

### 2.3. Histopathological evaluation

For microscopic evaluation, the tissues were sectioned at 0.4 mm intervals and embedded in paraffin wax. The paraffin wax was sliced of 4-micron thickness. After 24 h with alcohol fixation, hematoxylin eosin (HE) stain was applied to these tissues. The final forms of sections were examined under light microscope by 2 pathologists. They were blinded to the specimens. All groups were compared to each other according to presence of fibrosis, inflammation, and hyperemia-bleeding (Table 1). 

**Table 1:** Comparison of experimental groups according to the presence of histopathological features.

	Group PRn = 8	Group AVn = 8	Group PAVn = 8	Group Cn = 8	p
Inflammation n (%)	5 (62.5)	0	1 (12.5)	3 (37.5)	0.031
Hyperemia-bleeding n (%)	8 (100)	7 (87.5)	8 (100)	8 (100)	1.000
Cavernosal tissue healing with fibrosis n (%)	6 (75)	2(25)	2 (25)	3 (37.5)	0.162

PR = primary repair, AV = aloe vera, PAV= primary repair + aloe vera, C = control,

### 2.4. Statistical analysis

The data analyses were performed with SPSS Statistics v: 20.0 for Windows (IBM SPSS Inc., Chicago, IL, USA). Categorical variables were given as percentages and were analyzed with the Fisher’s exact test. Significance was set at p < 0.05.

## 3. Results

**Figure 1:**
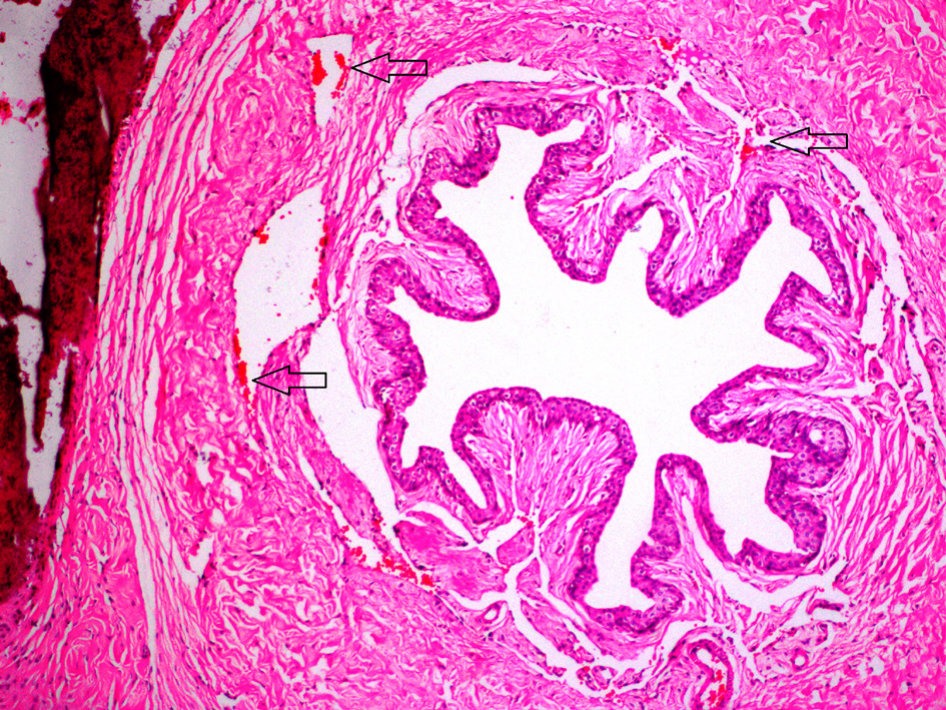
In group AV; mild hyperemia was predominant (arrows) (H&E, × 200).

**Figure 2:**
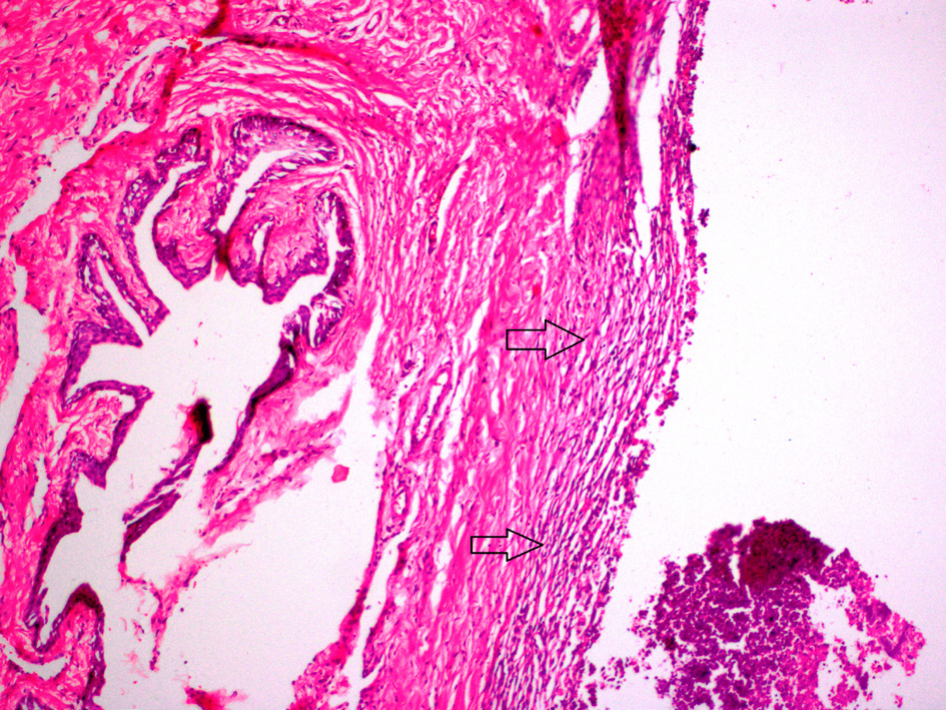
Inflammation was most seen in group PR (arrows) (H&E, × 200).

**Figure 3:**
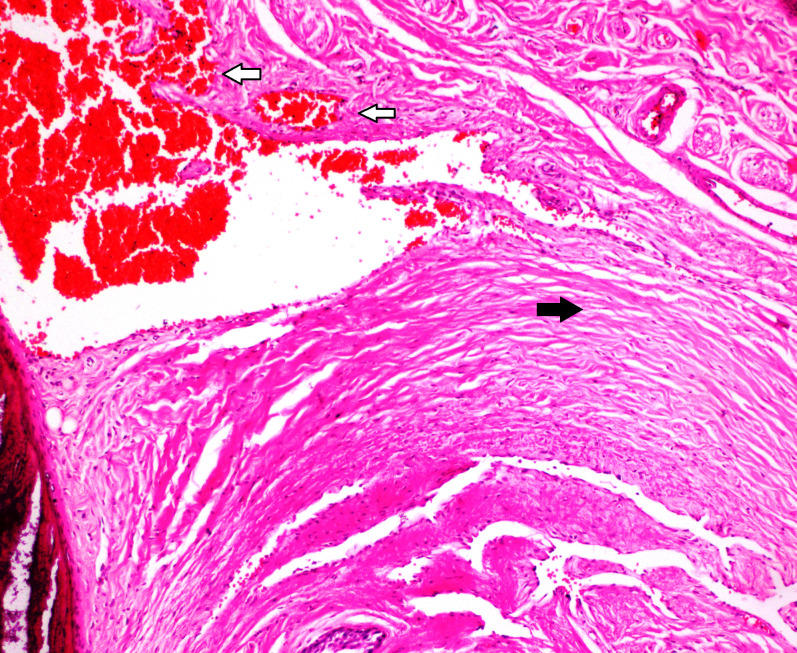
In group PAV; hyperemia-bleeding (white arrows) and mild fibrosis (black arrow) were observed (H&E, × 200).

**Figure 4:**
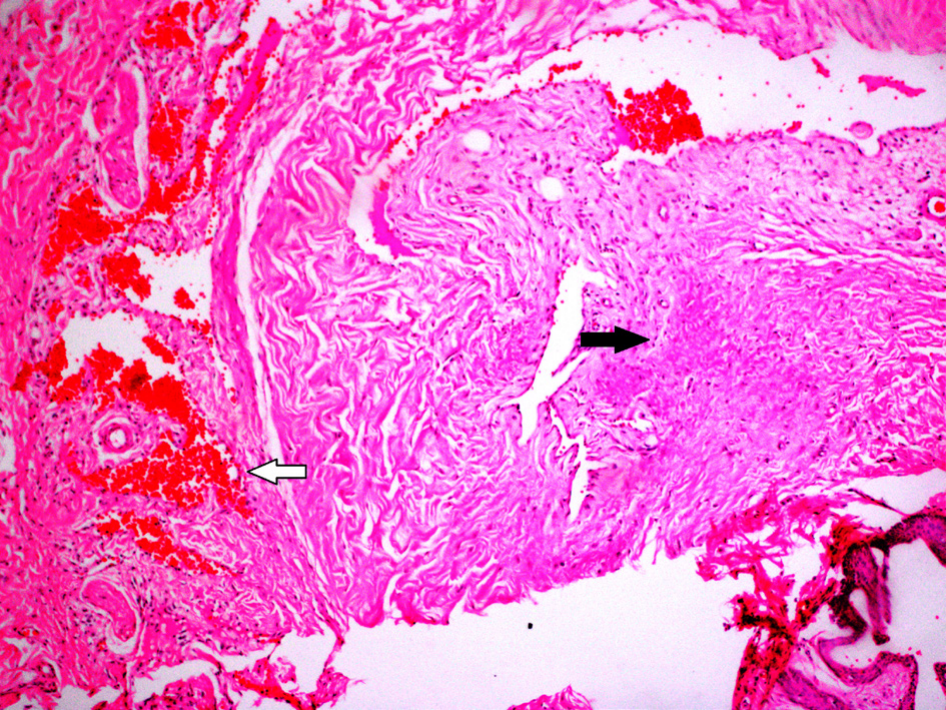
In group C; hyperemia-bleeding (white arrow) and fibrosis (black arrow) were observed (H&E, × 200).

In this study, no mortality was observed during three weeks after the procedures. Allergic reactions, urinary retention, and infection were not observed. In group AV, there were fibrosis in 2 (25%) rats and hyperemia-bleeding in 7 (87.5%) rats (Figure 1). Inflammation was not observed in group AV. In group PR; inflammation was seen in 5 (62.5%) rats and this score was higher than other groups (Figure 2). In 8 (100%) rats, there were hyperemia-bleeding features. Fibrosis was present in 6 (75%) rats. In group PAV, inflammation was observed in 1 (12.5%) rat. Hyperemia-bleeding and fibrosis were observed in 8 (100%) rats and 2 (25%) rats, respectively (Figure 3). In group C, hyperemia-bleeding was predominant feature. Hyperemia-bleeding was present in 8 (100%) rats and fibrosis was present in 3 (37.5%) rats (Figure 4). Inflammation was also observed in 3 (37.5%) rats. According to inflammation, no significant difference was observed between group C and group PR (p = 0.619). There was no inflammation in group AV and therefore, group AV had a better score than group PR (p = 0.026). In group PAV, inflammation was less seen than group PR, however, the difference was insignificant (p = 0.119). There was no difference between group AV and group PAV. In group C, 3 (37.5%) rats had inflammation and this score was not significantly different from group AV (p = 0.200) (Table 2).

**Table 2:** Comparison of experimental groups according to fisher’s exact test (p < 0.05).

		Inflammation	Hyperemia-bleeding	Cavernosal tissuehealing with fibrosis
		p	p	p
Group PR vs.	Group AV	0.026	1.000	0.132
	Group PAV	0.119	-	0.132
	Group C	0.619	-	0.315
Group AV vs.	Group PAV	1.000	1.000	1.000
	Group C	0.200	1.000	1.000
Group PAV vs.	Group C	0.569	-	1.000

Hyperemia-bleeding were seen in all groups with varying degrees and the difference between groups was insignificant (p = 1.000) (Table 1). In group AV and group PAV fibrosis was observed in 2 (25%) rats each and these groups had best scores for cavernous healing without fibrosis. However, when group PR was compared with group AV and group PAV, the differences in cavernous tissue healing with fibrosis were not significant (p = 0.132 and p = 0.132, respectively). Although fibrosis was observed two times more than group C in group PR, the difference was not statistically significant (p = 0.315) (Table 2).

The comparison of the experimental groups, according to histopathological parameters, revealed that in group AV all histopathological features were less seen than other groups. However, no statistical difference was observed between groups PAV and AV according to all features. In PAV group, only 1 rat (12.5%) had inflammation and this result made the group PAV the second best group.

## 4. Discussion

PF is a urological emergency, which is usually needed to be repaired immediately. It usually happens after a sexual intercourse (46%), forced flexion (21%) and masturbation (18%), rolling over in bed is rare (8.2%–9.5%) [16,17]. The rupture of the corpus cavernosum occurs at proximal penis over 60% of the patients [18]. Tunica albuginea is a thin structure. Its thickness is 2 mm during flaccidity. This thin layer’s thickness decreases to 0.25 to 0.50 mm during erections. Eighty percent of the ruptures are located in ventral side of the penis, because the thinnest region is on the ventral side including only a single layer [19]. For a physiologic simulation, in our study, we formed PF model at the proximal and anterior part of the rat penis. 

Today, the standard approach to treat PF is early surgical repair of tunica albuginea [20]. The purpose of early surgical restoration for providing anatomical and physiologic continuity in tunica albuginea is to prevent fibrosis that can lead to penile curvature. Previous experimental studies showed the contributing effects of tissue glue and hemostatic agents on the healing process of cavernosal tissue [13,14]. In this study, we specially aimed to explore a new adjuvant material for using after penile cavernosal surgery to prevent excessive inflammation and fibrosis because as we know from the literature some degree of scar formation on PF repair region was reported in 25% of patients [19]. Because of its well-known antiinflammatory, wound healing, and epithelization properties, we investigated topical application of aloe vera in the current study [21]. In the present study, fibrosis was observed in 6 (75%) rats and hyperemia-bleeding was detected in 8 (100%) rats in primary repair group. Small hemorrhages may develop in the tunica albuginea as a result of PF and surgical treatment. Therefore, in our study, hyperemia and bleeding were seen in almost all rats as a result of cavernosal trauma. 

Another parameter, inflammation was not observed in group AV. In PAV group, it was observed in only 1 (12.5%) rat. As we know from the literature, inhibition of TNF alpha and proinflammatory cytokines, including IL-6, improves cavernosal inflammation and functions [22]. According to us, the decreased rates of inflammation in groups AV and PAV were probably due to the antiinflammatory effect of aloe vera. It is effective in inhibiting inflammatory reactions by the inhibition of IL-6 and IL-8, the reduction of leukocyte adhesion, an increase of IL-10 levels, and decrease of TNF alpha levels [6]. Although, in group PAV, only 1 (12.5%) rat had inflammation, the difference between group PR and group PAV was not significant (p = 0.119). This score in group PAV might be due to suturing effect despite using aloe vera in group PAV. However, in our opinion, this result still can make AV as a clinically promising adjuvant local therapy after cavernosal surgeries. 

After the surgical repair of the PF, fibrosis of the corpus cavernosum might be attributed to the suture material as a result of healing process. In general, fibrosis is known as a result of an abnormal wound-healing and it is often the end stage of disorders caused by tissue injury and inflammation [23]. Fibrosis may lead to decreased elasticity and less efficient wound healing. Therefore, cavernosal tissue healing with fibrosis is not an optimal type of wound healing. In our study, local application of aloe vera was associated with decreased cavernosal fibrosis. In primary repair group (group PR), cavernosal tissue healing with fibrosis was observed in 6 (75%) rats. The fibrosis rate was decreased in aloe vera applied groups. Fibrosis was seen in 2 (25%) rats in group PAV and was seen in 2 (25%) rats in group AV. However, the differences between group PR and groups AV and PAV were not significant (p = 0.132 and p = 0.132, respectively). Although we did not observe a statistically significant difference in term of fibrosis, these results showed us that AV application may cause better wound healing without fibrosis. 

## 5. Conclusions

In conclusion, we evaluated histopathological effects of local sloe vera application after PF surgery. We detected that sloe vera can be helpful in and after cavernosal surgeries because of its antiinflammatory effect. Furthermore, we observed that the local application of sloe vera onto the PF region without closing with suture seems to be more beneficial than primary suturing of the corpus cavernosum. Further clinical studies evaluating the clinical use of aloe vera in PF repair are also needed.

## Informed consent

Ethical approval was obtained from the local ethical committee of University of Health Sciences Ankara Training and Research Hospital (Approval No:634, Date:20.08.2020).

## References

[ref1] (2017). Penile fracture epidemiology, diagnosis and management in Iran: a narrative review. Translational Andrology and Urology.

[ref2] (2020). Surgical reconstruction for penile fracture: a systematic review. International Journal of Impotence Research.

[ref3] (1999). Penile refracture. British Journal of Urology.

[ref4] (2021). The management of penile fracture: a review of the literature with special consideration for patients undergoing collagenase clostridium histolyticum injection therapy. Current Urology Reports.

[ref5] (2020). Surgical reconstruction for penile fracture: a systematic review. International Journal of Impotence Research.

[ref6] (2019). The effect of aloe vera clinical trials on prevention and healing of skin wound: a systematic review. Iranian Journal of Medical Sciences.

[ref7] (2009). Implications for degenerative disorders: antioxidative activity, total phenols, flavonoids, ascorbic acid, beta-carotene and beta-tocopherol in Aloe vera. Oxidative Medicine and Cellular Longevity.

[ref8] (2008). Evaluation of antioxidant, antinociceptive, and anti-inflammatory activities of ethanol extracts from Aloe saponaria Haw. Phytotherapy Research.

[ref9] (2009). Purification of aloe polysaccharides by using aqueous two-phase extraction with desalination. Natural Product Research.

[ref10] (2005). Chemical characterization of the immunomodulating polysaccharide of Aloe vera L. Carbohydrate Research.

[ref11] (2005). Identification of optimal molecular size of modified Aloe polysaccharides with maximum immunomodulatory activity. International Immunopharmacology.

[ref12] (2010). Effects of Scutellariae radix and Aloe vera gel extracts on immunoglobulin E and cytokine levels in atopic dermatitis NC/Nga mice. Journal of Ethnopharmacology.

[ref13] (2008). Effect of cyanoacrylic glue on penile fracture: an experimental study. Journal of Urology.

[ref14] (2009). Haemostatic and histopathological effects of ankaferd blood stopper, on penile cavernosal tissue in rats. International Journal of Hematology and Oncology.

[ref15] (2009). Effects of the application of Aloe vera (L.) and microcurrent on the healing of wounds surgically induced in Wistar rats. Acta Cirúrgica Brasileira.

[ref16] (2016). Penile fracture: a meta-analysis. Urologia Internationalis.

[ref17] (2013). Long-term treatment outcomes between surgical correction and conservative management for penile fracture: retrospective analysis. Korean Journal of Urology.

[ref18] (2015). Penile fracture: our experience in a tertiary care hospital. The World Journal of Men’s Health.

[ref19] (2018). Current management of penile fracture: an up-to-date systematic review. Sexual Medicine Reviews.

[ref20] (2019). Penile fracture and investigation of early surgical repair effects on erectile dysfunction. Urologia.

[ref21] (2020). Adding herbal extracts to silicone gel on post-sternotomy scar: a prospective randomised double-blind study. Journal of Wound Care.

[ref22] (2018). Depression induced by chronic stress leads to penile cavernosal dysfunction: protective effect of anti-TNF-α treatment. Canadian Journal of Physiology and Pharmacology.

[ref23] (2019). The mechanisms and potential of stem cell therapy for penile fibrosis. Nature Reviews Urology.

